# Coronary Microvascular Dysfunction and Hypertension: A Bond More Important than We Think

**DOI:** 10.3390/medicina59122149

**Published:** 2023-12-11

**Authors:** Marija Zdravkovic, Viseslav Popadic, Slobodan Klasnja, Andrea Klasnja, Tatjana Ivankovic, Ratko Lasica, Dragan Lovic, Drasko Gostiljac, Zorana Vasiljevic

**Affiliations:** 1 Clinic for Internal Medicine, University Clinical Hospital Center Bezanijska Kosa, 11000 Belgrade, Serbia; sekcija.kardioloska@gmail.com (M.Z.); slobodan.klasnja@gmail.com (S.K.); andrea.m93@gmail.com (A.K.); ivankovictatjana2@yahoo.com (T.I.); 2 Faculty of Medicine, University of Belgrade, 11000 Belgrade, Serbia; drlasica@gmail.com (R.L.); doctor@med.bg.ac.rs (D.G.); zoranavasiljevic47@gmail.com (Z.V.); 3 Clinic of Cardiology, Clinical Center of Serbia, 11000 Belgrade, Serbia; 4 Clinic for Internal Diseases Inter Medica, 18000 Nis, Serbia; lovicbdragan@gmail.com; 5 School of Medicine, Singidunum University, 18000 Nis, Serbia; 6 Clinic of Endocrinology, Diabetes and Metabolic Diseases, Clinical Center of Serbia, 11000 Belgrade, Serbia

**Keywords:** coronary microvascular dysfunction, hypertension, hypertensive heart disease, myocardial fibrosis, heart failure

## Abstract

Coronary microvascular dysfunction (CMD) is a clinical entity linked with various risk factors that significantly affect cardiac morbidity and mortality. Hypertension, one of the most important, causes both functional and structural alterations in the microvasculature, promoting the occurrence and progression of microvascular angina. Endothelial dysfunction and capillary rarefaction play the most significant role in the development of CMD among patients with hypertension. CMD is also related to several hypertension-induced morphological and functional changes in the myocardium in the subclinical and early clinical stages, including left ventricular hypertrophy, interstitial myocardial fibrosis, and diastolic dysfunction. This indicates the fact that CMD, especially if associated with hypertension, is a subclinical marker of end-organ damage and heart failure, particularly that with preserved ejection fraction. This is why it is important to search for microvascular angina in every patient with hypertension and chest pain not associated with obstructive coronary artery disease. Several highly sensitive and specific non-invasive and invasive diagnostic modalities have been developed to evaluate the presence and severity of CMD and also to investigate and guide the treatment of additional complications that can affect further prognosis. This comprehensive review provides insight into the main pathophysiological mechanisms of CMD in hypertensive patients, offering an integrated diagnostic approach as well as an overview of currently available therapeutical modalities.

## 1. Introduction

Hypertension represents a massive global health issue and is one of the most important cardiovascular risk factors. Hypertension-mediated organ damage (HMOD) is common in patients with severe or long-standing hypertension and is also prevalent in less severe hypertension, even in asymptomatic individuals with elevated blood pressure [1]. It is important to note that at any given blood pressure category above the normal or optimal, the presence of HMOD is associated with a 2- to 3-fold increase in the cardiovascular risk [2]. Up to 40% of newly diagnosed hypertensive patients already have HMOD, predominantly functional and structural alterations of heart, kidneys, eyes, brain, and peripheral arteries [3].

Hypertension is a well-established risk factor for the development of coronary microvascular dysfunction (CMD) [4]. The constant high pressure within the larger arteries can lead to damage and remodeling of the smallest arteries and arterioles in the microcirculation, capillaries, and venules, affecting their ability to regulate blood flow [5]. This leads to structural and functional remodeling of the coronary microcirculation, in which endothelial dysfunction is one of the most important pathogenetic mechanisms [6]. The endothelium plays a crucial role in regulating blood vessel tone and controlling blood flow. In hypertensive individuals, endothelial dysfunction significantly contributes to the development of CMD, which progressively leads to increased resistance in coronary microcirculation and limited blood flow, causing a reduced oxygen supply to the myocardium [7]. This is why the finding of myocardial ischemia as a result of CMD is relatively common in patients with hypertension, especially in patients with hypertensive heart disease (HHD). Many additional risk factors also contribute to the development of CMD in hypertensive patients, including metabolic syndrome, diabetes mellitus, hyperlipidemia, smoking, and others [8,9,10]. As the development of hypertensive heart disease progresses, left ventricular hypertrophy is more pronounced, consequently leading to more severe impairment of coronary microcirculation. These changes, accompanied by myocardial fibrosis, lead to an increased risk of heart failure with both preserved (HFpEF) and reduced ejection fraction (HFrEF) [11,12]. This is why coronary microvascular dysfunction significantly affects the morbidity and mortality of patients, demanding more purposeful diagnostic and therapeutic algorithms.

The purpose of this narrative review is to describe the relationship between coronary microvascular dysfunction and systemic hypertension, as well as its pathogenetic mechanisms, characteristics, and potential role in the development of adverse cardiovascular events, especially heart failure with preserved ejection fraction.

## 2. Pathogenetic Mechanisms of Coronary Microvascular Dysfunction

**Figure 1:**
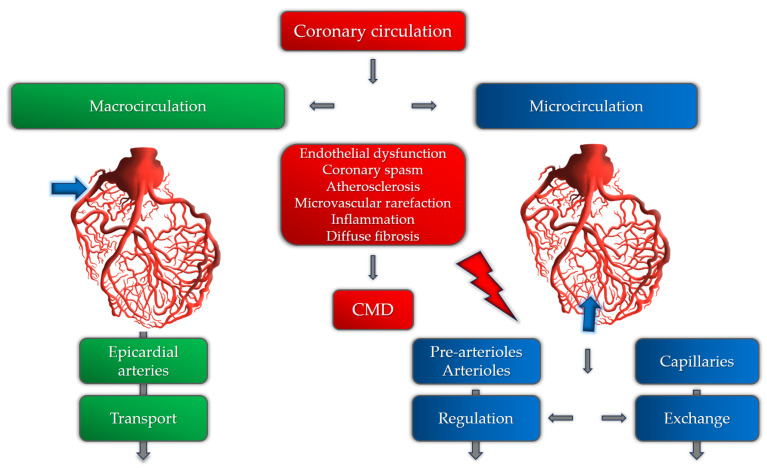
Coronary circulation and the role of different pathogenetic mechanisms involved in CMD.

The coronary microcirculation consists of pre-arterioles, arterioles, and capillaries. The main aim of coronary microvasculature is to match blood supply to myocardial oxygen consumption. Any increase in oxygen consumption leads to increased oxygen demands, consequently leading to an increase in myocardial blood flow (MBF). The main role in the control of myocardial blood flow is played by pre-arterioles and arterioles by controlling arterial diameter and tone. In coronary microvascular dysfunction, various mechanisms involved in this process are disrupted by several factors [Figure 1].

The mechanisms involved in CMD can be structural, functional, or a combination of both [13]. The main pathogenetic mechanisms of coronary microvascular dysfunction in patients with hypertension are still insufficiently researched. Until now, it has been postulated that the pathogenetic basis for the development of CMD involves a variety of mechanisms, including microvascular spasm, endothelial dysfunction, sympathetic over-activity, influence of female hormones, certain psychological disorders, and others [14,15]. These mechanisms are more likely to cause CMD in susceptible patients with hypertension, hyperlipidemia, obesity, or diabetes mellitus [16]. In patients with hypertension, the development of left ventricular hypertrophy and the subsequent development of myocardial fibrosis and diastolic dysfunction are important mechanisms of CMD due to several functional and anatomical changes in the microcirculation [17]. Maladaptive mechanisms in hypertension, perivascular fibrosis, and the thickening and rarefaction of small vessel walls, are responsible for increased microvascular resistance and inappropriate blood flow distribution [18]. Also, several functional mechanisms are described as causes of CMD in patients with hypertension, including reduced nitric oxide availability as the most important one [19,20]. It is shown that chronic renin–angiotensin system (RAS) over-activity, nicotinamide adenine dinucleotide phosphate oxidase, cyclooxygenase, xanthine oxidase, and uncoupled endothelial nitric oxide synthase (NOS), as sources of reactive oxygen species, are the main causes of NO deficiency [21]. Also, adrenergic activation and prolonged vasoconstriction can also lead to microvascular remodeling and rarefaction, causing ischemia and clinically manifested angina [22,23]. It is also important to note that certain studies registered these microvascular changes even in patients without elevated blood pressure, suggesting that microvascular dysfunction and remodeling can precede the onset and development of hypertension [24,25]. However, this cause–effect relationship needs further investigation.

### Microvascular Angina and Endothelial Dysfunction

Endothelial dysfunction is bi-directionally related to systemic hypertension. It has been shown that the endothelium controls vascular smooth muscle tone in response to various agents, as well as participating in the pathogenesis of hypertension by producing different mediators with systemic effects [26]. In patients with hypertension, endothelial dysfunction is mainly characterized by impaired nitric oxide synthesis and availability, as well as prostacyclin (PGI2) and endothelium-derived hyperpolarizing factor (EDHF) deficiency [27]. On the other hand, as a response to reactive oxygen species, increased production of endothelium-derived vasoconstrictors (mainly endothelin-1 and angiotensin-converting enzyme) has been observed [28]. This is subsequently associated with the development of vascular inflammation, vascular remodeling, and atherosclerosis. As a result, vasoconstrictive, pro-inflammatory, and pro-thrombotic mediators cause increased vasoconstrictive microvascular reactivity [29]. This process leads to both functional and structural changes in the microvasculature and the development of microvascular dysfunction. It is important to emphasize that CMD in patients with hypertension is not solely a result of hypertension but a multifactorial disease with a significant impact on cardiovascular morbidity and mortality.

## 3. Additional Risk Factors

### 3.1. Sex-Related Differences in Patients with Coronary Microvascular Dysfunction and Hypertension

Coronary microvascular dysfunction is more prevalent in women than in men [30]. Early works on estimating the sex-related differences in coronary microcirculation revealed lower coronary flow reserve (CFR) values in women, predominantly due to differences in resting coronary flow [31]. This is also in relation to different mechanisms involved with autonomic regulation and response to oxidative stress, adenosine, endothelin-1, and angiotensin II [32]. It is also notable that women have a smaller vessel size than men, which can contribute to lower CFR values [33]. Studies on cardiac magnetic resonance revealed specific differences, where women in comparison with men had fewer or no associations between the development of CMD and traditional risk factors, including hyperlipidemia, diabetes, smoking, and obesity [34]. This can mainly be the effect of ovarian hormone deficiency, as microvascular angina and estrogen deficiency in hypertensive women have demonstrated an association [35]. In the subgroup of both premenopausal and postmenopausal women with hypertension, ovarian dysfunction and consequent estrogen deficiency played a role in the pathogenesis of CMD [36]. Also, certain psychological factors can play an important role in the development of coronary artery disease, as well as CMD [37]. It is demonstrated that psychological stress induces endothelial dysfunction and vasomotor disorders more often in young women than in men [38].

### 3.2. Metabolic Syndrome

Metabolic syndrome includes a cluster of conditions such as central obesity, dyslipidemia, high blood pressure, and impaired fasting glucose, all related to an increased cardiovascular risk [39]. Several studies have demonstrated a correlation of different variables with the presence of microvascular dysfunction in these patients, including age, sex, pulse pressure, fasting glucose, hemoglobin A1c (HbA1c), total cholesterol, low-density lipoprotein (LDL)-cholesterol, estimated glomerular filtration rate (eGFR), and albuminuria [40]. Among patients with hypertension, it is shown that patients with metabolic syndrome have a more severe form of CMD than those without metabolic syndrome. Sucato et al. demonstrated that these patients had worse coronary perfusion than patients with diabetes mellitus [41].

### 3.3. Obesity

Obesity has been linked to chronic metabolic disorders, resulting in poor clinical outcomes. Increased oxidative stress, sympathetic nervous system over-activity, and low-grade systemic inflammation are the main mechanisms of coronary microvascular dysfunction in obese individuals [42]. The metabolic activity of adipose tissue, as well as different cytokines and adipokines, are responsible for reduced NO-mediated dilatation, changed endothelial and smooth muscle-dependent vasoregulation mechanisms, and altered vasomotor control. In patients with hypertension, additional volume overload and cardiomyocyte hypertrophy contribute to vascular remodeling. The thickness of epicardial fat tissue, which reflects visceral adiposity rather than general obesity, is also predictive of an impaired coronary vasodilator capacity [43]. A study by Bajaj et al. demonstrated that obese patients with CMD have a 2.5-fold higher risk of developing adverse clinical events [44]. It has been shown that patients with central obesity have lower values of myocardial blood flow than patients with excess weight and no central obesity. This is important to notice, as cardiovascular risk estimation based on waist-to-height ratio and the presence of central obesity becomes more prevalent than that based on BMI, which is recognizable especially in the case of “obesity paradox” and patients with heart failure [45]. Considering the variety of metabolic disorders in the obese population, weight loss and intensified risk-factor control in patients with CMD play an important role in improving angina symptoms, as presented in a study by Bove et al. [46].

### 3.4. Diabetes Mellitus

The key mechanisms of CMD in patients with diabetes are impaired coronary arteriole vasomotion, including impaired endothelial-mediated vasodilation, hypoxia-induced vasodilation, and myogenic response [47]. It has been shown that hyperglycemia and insulin resistance play central roles in the development of CMD by leading to oxidative stress, inflammatory activation, and endothelial dysfunction [48]. In the later stage of diabetes, structural changes occur. Thickening of the capillary basement membrane and of the arteriole wall results in luminal narrowing, perivascular fibrosis with focal constriction, and capillary rarefaction. These mechanisms lead to increased coronary microvascular resistance and reduced coronary flow reserve and can cause myocardial ischemia [49]. CMD is common in patients with diabetes and can be present with or without the finding of significant epicardial coronary artery disease. It has been shown, by certain studies, that more than 70% of patients with type 2 diabetes mellitus have CMD, which can seriously affect future cardiovascular events and prognosis, especially in those with acute myocardial infarction and heart failure [50].

### 3.5. Hypercholesterolemia

Numerous studies have shown that hypercholesterolemia leads to an inflammatory response within the microvasculature, decreased availability of nitric oxide, and increased production of reactive oxygen species (ROS) [51,52]. Endothelial dysfunction and capillary rarefaction are the two most important mechanisms, leading to severe microvascular impairment in different organs and provoking glomerulopathy-induced kidney dysfunction and hypertension, reduction in coronary flow reserve leading to coronary microvascular dysfunction, and hepatic dysfunction, as in non-alcoholic fatty liver disease [53]. It has been shown that the role of specific vasoactive substances is related to both hypercholesterolemia and hypertension, as well as the development of CMD, predominantly endothelium-dependent microvascular dysfunction. This is representative of the pathway of thromboxane A2, which has an important role in platelet aggregation, vasoconstriction, and proliferation [54]. Certain studies demonstrated that patients with uncontrolled hypertension and hypercholesterolemia had increased thromboxane A2 production, which resulted in excessive vasoconstriction, arteriolar remodeling, and capillary rarefaction [55].

### 3.6. Obstructive Sleep Apnea

Obstructive sleep apnea (OSA) is a condition linked to increased cardiovascular morbidity and mortality [56]. Repetitive episodes of hypoxemia lead to the excessive production of reactive oxygen species, the development of low-grade inflammation, and endothelial dysfunction. It has been shown that patients with moderate to severe obstructive sleep apnea have lower values of CFR [57]. However, the exact influence of OSA on the development and progression of CMD is hard to observe, as these patients usually have several other risk factors related to CMD, including hypertension, diabetes mellitus, obesity, and hyperlipidemia.

### 3.7. Smoking

Cigarette smoke is known as the exertion factor with the most detrimental effects on the endothelium, especially the coronary endothelial system [58]. Various toxic components can cause severe endothelial damage, reduce hyperemic coronary blood flow velocity, and provoke the development of microvascular dysfunction. Regarding the presence of CMD, Gullu et al. demonstrated that smokers without obstructive epicardial coronary disease had significantly lower values of coronary flow velocity reserve (CFVR) than the control group [59]. On the other hand, even in patients with epicardial coronary artery disease, smoking was associated with impaired invasively derived indices of coronary microvascular dysfunction, which can additionally contribute to a worse prognosis [60].

## 4. Diagnostics for Coronary Microvascular Dysfunction in Patients with Hypertension

In recent years, several important diagnostic algorithms have been presented regarding CMD that aim to integrate both non-invasive and invasive modalities [61]. The diagnostic algorithm in patients with suspected CMD starts with the exclusion of significant epicardial coronary artery disease. Although CMD can be present in patients with obstructive CAD, the presence of CMD in the absence of obstructive CAD is extremely important to diagnose, especially in patients with additional risk factors for the development of adverse cardiovascular events, primarily heart failure [62]. In patients with microvascular angina, non-invasive diagnostic imaging modalities, primarily echocardiography and cardiac magnetic resonance (CMR), are important for the evaluation of alternative causes of chest pain, including structural and inflammatory conditions [63]. Patients with a negative coronary angiogram, a positive stress test for myocardial ischemia, and additional risk factors for the development of CMD (especially those with hypertensive heart disease) should be considered for non-invasive and invasive investigation of CMD.

### 4.1. Non-Invasive Diagnostics

#### 4.1.1. Echocardiography

Conventional echocardiographic stress tests have limited utility in the diagnosis of CMD, as significant inter-observer variability is present in cases with low to moderate ischemia burden, resulting in hypokinesia [64]. The use of echocardiography in detecting coronary microvascular dysfunction mainly relies on myocardial contrast echocardiography and the estimation of myocardial blood flow (MBF) or coronary flow velocity reserve using pulsed-wave Doppler sampling of the proximal left anterior descending coronary artery. Nowadays, CFVR has higher diagnostic accuracy and better correlation with intracoronary Doppler wire-based techniques, especially in patients with HFpEF, as demonstrated in the PROMIS-HFpEF trial [65]. Numerous studies have investigated the prognostic significance of CFVR in patients with hypertension, demonstrating an impairment in microvascular vasodilatation capacity even in the early stages of the disease [66,67]. The study by Volz et al. showed that CFVR was significantly lower in patients with resistant hypertension than in individuals with non-resistant hypertension, indicating a more severe impairment of coronary microvascular function that could account for the increased risk of adverse outcomes [66]. The main disadvantages of MBF assessment of CFVR are the presence of artifacts and high inter-observer variability, especially in obese patients and patients with lung disease. However, these methods can be helpful as inexpensive methods in the initial assessment of patients with CMD. In addition to its significant role in the diagnosis of obstructive coronary artery disease, strain assessment is becoming equally important in patients with CMD [68]. Aside from CFRV, novel protocols of stress echocardiography incorporate the estimation of global longitudinal strain in rest and peak stress to increase sensitivity and specificity of this estimation [69]. A study by Jovanovic et al. demonstrated that resting, peak, and ΔLVGLS were all significantly impaired in female patients with coronary microvascular dysfunction and slow coronary flow [70].

#### 4.1.2. Computerized Tomography (CT)

The role of CT coronary angiography is to primarily exclude the existence of significant epicardial coronary artery disease. Recent technical and software advancements provide the possibility to follow the first pass of contrast through the myocardium at frequent intervals and estimate the absolute myocardial flow. Two types of CT myocardial perfusion protocols can be performed, static and dynamic. Static CT myocardial perfusion requires a lower amount of radiation and prospective ECG gating. However, only qualitative and semiquantitative evaluation is possible with this technique. Dynamic CT perfusion allows the estimation of myocardial perfusion in different layers of the myocardium and a complete quantitative myocardial blood flow evaluation, providing evidence of reduced subendocardial perfusion in patients with CMD [71]. Novel techniques combining CTA-derived FFR and estimation of myocardial perfusion can provide an accurate anatomical and functional assessment of both the myocardium and the coronary circulation within one examination, which can be significant, especially in patients with hypertensive heart disease [72]. Studies that investigated myocardial perfusion and coronary-volume-to-left-ventricular-mass ratio showed promising results in diagnosing patients with CMD [73]. However, the results in patients with hypertension are controversial. The study by van Rosendal and colleagues demonstrated that patients with hypertension and increased left ventricular (LV) mass did not have reduced coronary vascular volume that could be associated with the presence of abnormal perfusion reserve [74]. This can also be a result of predominantly functional impairment of coronary microcirculation, as well as a lack of the estimation of coronary vasodilator reserve.

#### 4.1.3. Single-Photon Emission Computed Tomography (SPECT)

With recent advancements in high-sensitivity cardiac cameras and radiotracers, dynamic SPECT found its place in the quantification of myocardial blood flow and the assessment of CMD. Nowadays, iodinated rotenone compounds and solid-state, high-sensitivity cadmium–zinc–telluride detectors can detect the first-pass blood perfusion of a tracer and its extraction into the myocardium. This allows the quantification of myocardial blood flow and myocardial perfusion reserve with better accuracy and fewer artifacts. [75]. This protocol results in better spatial resolution and higher sensitivity, resulting in shorter acquisition time and lower radiation exposure. Zhang et al. demonstrated that quantitative SPECT analysis of myocardial blood flow provides prognostic value in patients with ischemia and no obstructive coronary artery disease (INOCA) [76]. However, as the diagnostic and prognostic significance of SPECT is still under PET and CMR, it can allow clinically useful measurements in the absence of previously mentioned modalities.

#### 4.1.4. Positron Emission Tomography (PET)

The main advantages of PET in the estimation of CMD are global and regional measurements of perfusion, quantitative MBF, and function, both under stress and at rest. By estimating myocardial perfusion during rest and stress, it can accurately estimate myocardial perfusion reserve (MPR), a value that has an excellent correlation with invasive modalities and also with adverse outcomes [77]. As it can estimate both epicardial and microvascular coronary distribution, PET can improve risk stratification for patients being investigated for ischemia. Studies of patients with hypertension revealed that the “endogen” type of CMD, predominantly related to alterations in resting myocardial blood flow, is more prevalent in these patients [78]. High radiation exposure and cost are the main disadvantages of this method. In comparison to cardiac magnetic resonance, PET lacks the possibility to additionally provide a sophisticated myocardial tissue characterization.

#### 4.1.5. Cardiac Magnetic Resonance (CMR)

**Figure 2:**
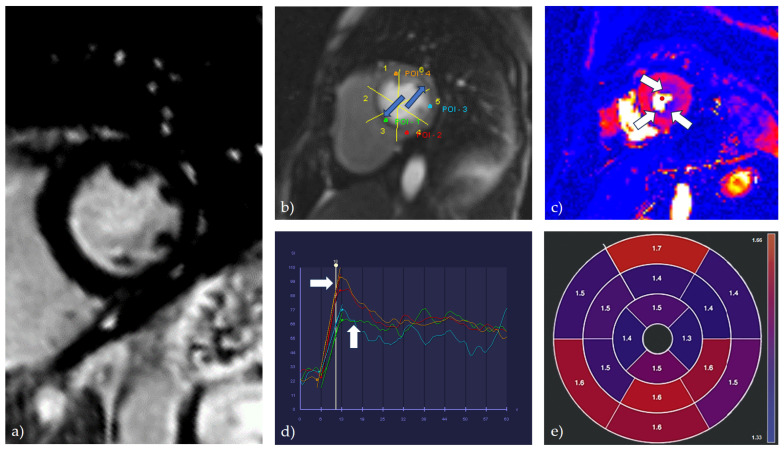
A combination of qualitative, semiquantitative, and quantitative methods for the evaluation of CMR stress perfusion study in a patient with coronary microvascular dysfunction. (**a**) LGE PSIR sequence, short axis view; showing the absence of LGE phenomenon; (**b**) qualitative analysis of stress perfusion; a global subendocardial perfusion defect is observed (marked by blue arrows); (**c**) perfusion map during stress perfusion study, short axis section, medial level; a global subendocardial perfusion defect is observed (marked by white arrows); (**d**) semiquantitative analysis (flow/time curve), short axis section, medial level; the perfusion curves indicate a global perfusion defect in the subendocardial layers of the myocardium (green and blue curves) in comparison to the subepicardial layers (red and orange curves) (marked by white arrows); (**e**) quantitative analysis of stress perfusion; diffusely reduced normalized values of myocardial perfusion reserve (MPRI) are observed.

Cardiac magnetic resonance has an important place in cardiac diagnostics, considering that it is a non-invasive method during which, with high specificity and sensitivity, the existence of both significant epicardial obstructive coronary disease and coronary microvascular dysfunction can be confirmed or excluded. Diagnostics of coronary microvascular dysfunction via CMR can be established by analyzing myocardial perfusion during the stress test in comparison with myocardial perfusion at rest, which actually evaluates the vasodilatory flow reserve [79]. During the stress perfusion test, various vasodilator agents can be used, including adenosine, regadenoson, or dipyridamole. Stress CMR accurately assesses myocardial ischemia, myocardial viability, and cardiac function, all in one examination. Methods within cardiac magnetic resonance to evaluate the existence of coronary microvascular dysfunction can be qualitative and quantitative [Figure 2]. A qualitative method of assessment includes visual evaluation of the perfusion during stress, whereby a characteristic diffuse subendocardial perfusion defect is observed. The drawback of the qualitative evaluation of the stress perfusion study is the extremely low sensitivity of only 41% and the inability to clearly differentiate between patients who have a pronounced degree of coronary microvascular dysfunction and patients who have multi-vessel CAD, which can also cause a diffuse subendocardial defect in perfusion [80]. If coronary angiography was not performed before the stress perfusion test, in the differentiation of coronary microvascular dysfunction and obstructive coronary disease, late gadolinium enhancement (LGE) sequences can be helpful, on which the zones of the LGE phenomenon are not registered in patients with microvascular dysfunction. Novel CMR diagnostic modalities, myocardial tissue mapping, and extracellular volume fraction (ECV) are important in estimating the presence and degree of interstitial fibrosis, which can be significant in risk stratification, especially in patients with hypertension who have left ventricular hypertrophy, diastolic dysfunction, and consequently an increased risk of HFpEF [81].

Semiquantitative and, especially, quantitative methods of evaluation of stress perfusion are used for definitive assessment. Quantitative methods of assessing coronary microvascular dysfunction can, in addition to establishing a diagnosis, evaluate the severity of the disease, as well as monitor the effect of different therapeutic modalities. New sophisticated and fully automated CMR methods for the analysis of myocardial perfusion enable high diagnostic accuracy, strong prognostic significance, and complete independence from the level of staff training [82]. The basic parameter for the analysis is the value of the blood flow through the myocardium (myocardial blood flow—MBF), which is analyzed both at rest (rest perfusion) and under stress (stress perfusion). Patients with global stress MBF below 2.25 mL/g/min without visual defects in perfusion are likely to have coronary microvascular dysfunction [83]. The difference in myocardial blood flow at rest and under stress represents the myocardial perfusion reserve (MPR), whose indexed value (MPRI) is the most sensitive parameter in the diagnosis of coronary microvascular dysfunction [84]. The accuracy of this method can be significantly increased by analyzing the myocardial perfusion reserve in the subendocardial layer (MPRendo), bearing in mind that the subendocardial layer of the myocardium is the most sensitive to the existence of ischemia [85]. The values of these parameters can be fully evaluated and quantified using pixelated perfusion maps at the level of individual segments according to the 16-segment model of the left ventricle. This kind of analysis makes it possible to establish a diagnosis with high sensitivity and specificity and also to differentiate the existence of obstructive coronary disease from coronary microvascular dysfunction. Clinically relevant values of the above-mentioned parameters for the diagnosis of coronary microvascular dysfunction can be registered even in the absence of qualitative changes in perfusion. In studies that used a fully quantitative assessment of stress perfusion to diagnose CMD, an excellent correlation was shown with the values of invasively measured coronary flow parameters (dominantly with the value of the coronary flow reserve—CFR) and with the value of the index of microvascular resistance (IMR) [86,87]. In terms of clinical outcomes, stress MBF and MPR/MPRI have been shown to be associated with serious adverse cardiovascular events and mortality [88].

Non-contrast-based CMR techniques for perfusion estimation are the future of CMD diagnostics as they are more sensitive and have even higher diagnostic accuracy than today’s widely available techniques. They are based on the principle of estimating myocardial tissue oxygenation by specific protocols or comparing the changes in myocardial native T1 time during the rest and stress perfusion study [89]. These techniques can overlook different limitations of conventional techniques, including imaging artifacts, long scan time, inter-observer variability, problems with the absolute quantitation of myocardial blood flow, and restricted use in patients with chronic kidney disease.

Advantages and disadvantages of non-invasive modalities in the estimation of CMR are presented in Table 1.

### 4.2. Invasive Diagnostics

The invasive modalities in the diagnostics of CMD are mainly based on the estimation of coronary blood flow. Coronary blood flow can be estimated by Doppler (measuring coronary flow velocity) or thermodilution (measuring cold bolus transit time), each with a different sensor-tipped intracoronary guidewire [90]. In regard to the endothelium function, coronary blood flow can be estimated in response to adenosine (non-endothelium-dependent function) or in response to acetylcholine to evaluate the presence of vasospastic angina (endothelium-dependent function). CFR values (the ratio of the maximal or hyperemic flow to the resting flow) of less than 2.0–2.5 (thermodilution) or 2.5 (Doppler) in the absence of epicardial obstructive coronary artery disease indicate the presence of coronary microvascular dysfunction [91]. The ratio between myocardial perfusion reserve and flow can be used to calculate coronary microvascular resistance (CMR). In the thermodilution-based method, the index of microvascular resistance (IMR) with a cut-off value of >25 is significant for confirming the presence of CMD, while in the Doppler-based technique, the resulting index is called hyperemic microvascular resistance (hMR), with the cut-off value of ≤2.5 mmHg/cm/s [92,93]. Regarding endothelium-dependent microvascular dysfunction, the diagnosis can be made if there is an increase of less than 50% in coronary blood flow, accompanied by ischemic ECG changes and angina symptoms, and in the absence of epicardial vasoconstriction. It is important to have in mind that patients with CMD may have both endothelium-dependent and -independent types of microvascular dysfunction. Studies evaluating the invasive indices of CMD in patients with HFpEF revealed abnormalities in coronary flow and resistance [94]. The study by Dryer et al. revealed that HFpEF patients had lower CFR and higher IMR values than the control group. These patients were also older and had higher values of NT-proBNP and higher left ventricular end-diastolic pressure, while 93% of them had hypertension as one of the comorbidities [95]. 

**Figure 3:**
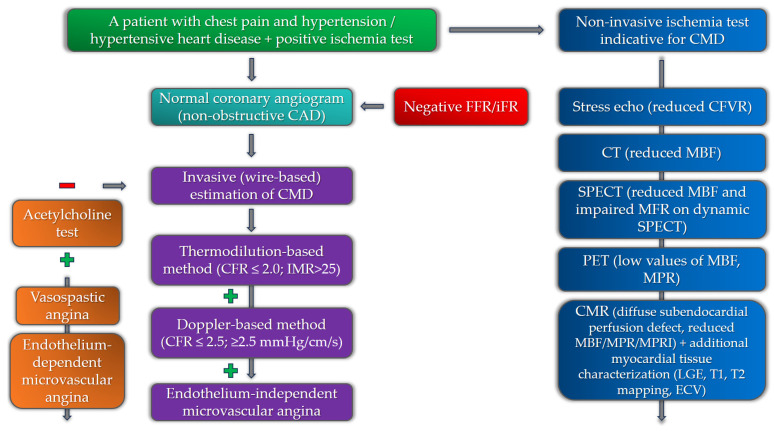
Diagnostic algorithm for the estimation of coronary microvascular dysfunction among hypertensive patients with chest pain (negative and positive symbols correspond to a negative or positive test in the diagnostic algorithm).

The diagnostic algorithm for the estimation of CMD among patients with chest pain and hypertension, involving both non-invasive and invasive modalities, is presented in Figure 3.

Considering the variety of imaging modalities in diagnostics for CMD, it is notable to mention that in patients with hypertension, the indices of arterial stiffness are independently related to microvascular dysfunction [96,97,98]. A recent study by Aursulesei Onofrei et al. demonstrated a predictive value of the subendocardial viability ratio (SEVR), also known as the Buckberg index, in hypertensive patients with CMD. This parameter of arterial stiffness, which represents an index of myocardial oxygen supply and demand, is significant in the assessment of long-term cardiovascular risk and is independently associated with age, abdominal circumference, and Framingham risk score [99].

## 5. Coronary Microvascular Dysfunction, Hypertension, and HFpEF

**Figure 4:**
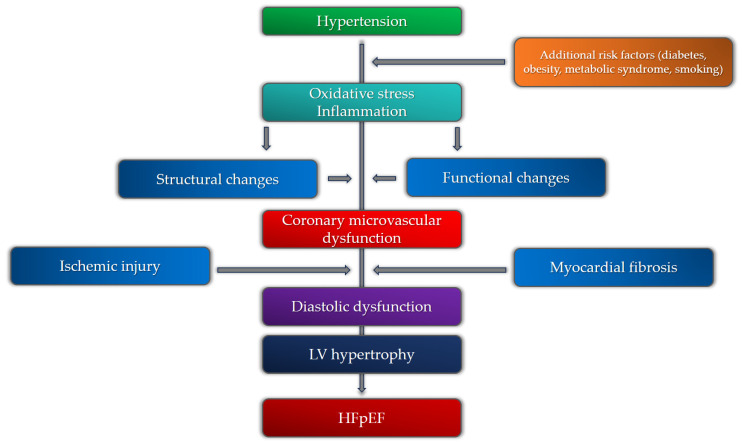
Pathophysiological mechanisms of heart failure with preserved ejection fraction (HFpEF) in relation to coronary microvascular dysfunction and hypertension.

Recent studies that researched the pathophysiology of HFpEF and the role of CMD revealed that, across various studies, 40–86% of patients with HFpEF have coronary microvascular dysfunction, proven by both non-invasive and invasive diagnostic modalities [100,101]. It is still uncertain whether CMD is a cause or a consequence of HFpEF. Since myocardial interstitial and focal fibrosis is one of the main mechanisms in HFpEF responsible for increased myocardial stiffness, it is believed that CMD and its consequences are at the core of HFpEF pathophysiology, mostly due to chronic microvascular inflammation [102]. The emerging role of inflammation in the development of HFpEF has been the subject of numerous studies in recent years. In patients with hypertension, inflammation is driven mainly by oxidative stress, inducing hypertension-related vascular aging through various mediators [103]. This process is shown to be one of the main mechanisms in the development and progression of HFpEF. Kanagala et al. demonstrated that CMD is an independent predictor of all-cause mortality and heart failure hospitalizations in patients with HFpEF [104]. It is important to note that a variety of other parameters were found to correlate with CMD and HFpEF, including age, heart rate, diastolic blood pressure, hemoglobin, urea, creatinine, eGFR, BNP, usage of loop diuretics, and increased LV filling pressures. Hypertension is one of the most important factors for the development of endothelial dysfunction and the promotion of pro-hypertrophic and pro-fibrotic signaling, thus directly increasing the risk for the development of CMD, diffuse and focal fibrosis, and HFpEF [105]. It has been shown that a significant number of patients with HFpEF exhibit hypertension as a comorbidity (up to 90%) [106]. The presence of CMD and hypertension, or more precisely, hypertensive heart disease, have prognostic significance in patients with HFpEF. Extracellular volume fraction, a marker of interstitial fibrosis assessed by cardiac magnetic resonance, is one of the most important parameters to discriminate between HHD and HFpEF. The amount of interstitial fibrosis that clinically correlates with significant LV stiffness, the development of HFpEF, and the transition from HHD to HFpEF is a value of ECV of 31.2%. This value can discriminate between HFpEF and HHD with 100% sensitivity and 75% specificity [107]. One more parameter derived from non-invasive diagnostic modalities that can differentiate between HHD and HFpEF is the global longitudinal strain (GLS). In hypertensive heart disease and in HFpEF, fibrosis involves the myocardial mid-wall, where circumferential shortening fibers are located, which is why global circumferential strain (GCS) is affected before longitudinal shortening. It has been found that GLS is significantly more depressed in patients with HFpEF than in patients with HHD, marking it as a more powerful prognostic marker in HFpEF [108]. One of the possible explanations could be the more pronounced focal, and especially interstitial, fibrosis in HFpEF patients as a consequence of advanced stages of CMD and LV hypertrophy. However, the exact relationship between all these clinical entities is yet to be determined. The cause-and-effect relationship between hypertension, numerous risk factors, and CMR in the development of HFpEF is presented in Figure 4.

## 6. Coronary Microvascular Dysfunction, Hypertension, and Atrial Fibrillation

As previously mentioned, myocardial fibrosis is one of the main consequences of both hypertensive heart disease and coronary microvascular dysfunction and is also an important pathophysiological mechanism of HFpEF. Cardiac magnetic resonance studies demonstrated the presence of myocardial fibrosis not only in the LV myocardium but also in the left atrium, subsequently increasing the risk of atrial fibrillation occurrence [109]. It is notable that, aside from being the most prevalent sustained arrhythmia in clinical practice, atrial fibrillation is particularly common in patients with HFpEF [110]. Although there is a lack of evidence on the exact relationship between CMD and AF, it is proposed that impaired myocardial perfusion in patients with CMD causes atrial remodeling and electrical instability, thus facilitating the occurrence of AF in patients with CMD. Recent studies evaluating the presence and impact of AF in patients with HFpEF revealed that AF is present in 79% of patients with HFpEF [111]. Among patients with AF and HFpEF, more than 90% of patients have impaired invasively derived values of CFR, indicating the presence of CMD. It is important to underline that in these patients, hypertension was significantly more prevalent, contributing to the development of CMD, AF, and HFpEF. Based on the above, it is important to search for CMD in patients with hypertension and atrial fibrillation, as these patients have an increased risk of developing HFpEF.

## 7. Management of Coronary Microvascular Dysfunction in Patients with Hypertension

Having in mind the variety of pathophysiological mechanisms and different clinical phenotypes, the management of coronary microvascular dysfunction is a challenging task. It is mainly a combination of pharmacological treatment and lifestyle modification, although, in the last few years, several interventional techniques have appeared as potential therapeutic solutions. Lifestyle interventions, including smoking cessation, weight loss, regular exercise, and improved nutrition, have demonstrated positive effects on microvascular function [112,113]. It is shown that the optimization of underlying diabetes mellitus and hyperlipidemia, and also the treatment of hypertension, as one of the most important risk factors, is beneficial in patients with CMD [114]. Early and continuous regulation of hypertension in patients with CMD is significant, as it can slow down the occurrence and progression of several subclinical and clinical entities such as left ventricular hypertrophy, interstitial myocardial fibrosis, and diastolic dysfunction. This can reduce the ischemic burden, improve symptoms, and reduce the risk of adverse events, especially HFpEF. AEC inhibitors, angiotensin receptor blockers (ARB), calcium channel blockers, and beta blockers with vasodilatory properties have substantial effects on improving microvascular perfusion [115,116,117]. Regarding the effects of ACE inhibitors, it is shown that certain medications can also slow down and even reverse reactive interstitial fibrosis, which is important in patients with hypertension [118]. The ongoing trial regarding the interventional treatment of hypertension (renal denervation) tends to suggest the positive effects of this procedure on patients with hypertension-related microvascular dysfunction, although the results of previous studies were controversial [117]. Considering the already proven positive effects of renal denervation on cardiac morphology and function, the additional effects on the improvement of microvascular function can be helpful in preventing both HFpEF and HFrEF [119]. Interventional procedures for the treatment of microvascular angina have been under development in recent years with promising results. The implantation of a coronary sinus reducer, which leads to a significant reduction in vascular resistance in the subendocardium, showed positive effects on angina symptom relief in patients with CMD [120]. Future studies should demonstrate the overall clinical benefit of this procedure in everyday practice.

## 8. Prognosis

Recent studies that investigated the prognostic significance of invasively derived indices of CMD revealed that depressed CFR was associated with an increased risk of cardiovascular death and heart failure admission, while elevated IMR alone still has a limited prognostic value [121]. It is still unclear why IMR has uncertain prognostic significance in patients with preserved CFR. However, one of the possible explanations can be that impaired IMR value can be an earlier indicator of CMD in the subclinical phase of the disease, with dominant functional alterations of the microcirculation. On the other hand, depressed CFR is more significant in the clinical phase of the disease, reflecting both functional and structural alterations, and is more associated with clinical outcomes in these patients. Non-invasive estimation of myocardial perfusion seems to have an additional prognostic significance. The greatest number of studies refer to CMR and PET as the two most important non-invasive modalities. In PET studies, there was a positive correlation with clinical outcomes in the group of patients with both epicardial and microvascular coronary artery disease, as well as with CMD solely [122]. The reduction of myocardial flow reserve was associated with the incidence of major adverse cardiovascular events (MACE) in both of these groups. The study by Murthy et al. demonstrated that there was a 3-year cardiac mortality rate of 8% in patients with impaired MFR, among which over 80% had hypertension as a comorbidity [123].

Quantitative CMR methods of estimating myocardial perfusion demonstrated a significant correlation with major adverse cardiovascular events. The value of MPRI (myocardial perfusion reserve index) below the optimal predictive threshold value of 1.47 was related to a three-fold increased risk of having MACE in the 5-year follow-up. It is important to underline that hypertension, alongside MPRI value, was also a significant predictor of poor prognosis in these patients, indicating an important mutual relationship between microvascular angina and hypertension [124].

## 9. Future Perspectives

A more integrated algorithm of CMD diagnostics, especially in symptomatic patients and patients with increased risk of HFpEF, is mandatory. This is important not only to control symptoms but also to minimize the possibility of future adverse cardiovascular events. Investigating the relationship between different clinical entities, especially CMD, myocardial fibrosis, hypertensive heart disease, and HFpEF, will be helpful in the proper identification of patients at risk and also to guide further development of different therapeutic modalities.

## 10. Conclusions

Coronary microvascular dysfunction is a clinical entity linked with various risk factors that significantly affect cardiac morbidity and mortality. Hypertension, one of the most important, causes both functional and structural alterations in the microvasculature, promoting the occurrence and progression of microvascular dysfunction. CMD is also related to several hypertension-induced morphological and functional changes in the myocardium in the subclinical and early clinical stages. This indicates the fact that CMD, especially if associated with hypertension, is a subclinical marker of end-organ damage and heart failure, particularly that with preserved ejection fraction. This comprehensive review provides an integrated diagnostic approach for patients with hypertension and suspected CMD, as well as an overview of current therapeutical modalities in order to reduce the burden of this emerging condition.

## Figures and Tables

**Table 1:** Characteristics of non-invasive imaging modalities in the evaluation of CMD.

Diagnostic Modality	Parameter	Advantages	Disadvantages
Echocardiography	CFRV	Low cost Low risk No radiation exposure	Obstructive CAD needs to be excluded Limited to LAD region Limited data in CMD Needs extensive training
CT coronary angiography and cardiac perfusion	MBF	Evaluation of coronary anatomy and perfusion Evaluation of both epicardial (angiography + FFR-CT angio) and microvascular territory	Radiation exposure Limited in chronic kidney disease Limited absolute MBF quantification Overestimation of MBF Limited data in CMD
PET	MPR, MBF	Gold standard Global evaluation of microvascular function Low radiation Good clinical correlations	Obstructive CAD needs to be excluded Limited availability Limited spatial resolution High costs Lack of sophisticated tissue characterization
CMR	MBF, MPR, MPRI	No radiation exposure Excellent spatial resolution Evaluation of all coronary territories Tissue characterization (myocardial mapping, ECV) Risk stratification	High costs Limited availability Obstructive CAD needs to be excluded Contraindicated in patients with severe kidney disease, non-MRI-conditional devices, claustrophobia

CAD—coronary artery disease; CRFV—coronary flow velocity reserve; CMR—cardiac magnetic resonance; CT—computerized tomography; LAD—left anterior descending artery; MBF—myocardial blood flow; MPR—myocardial perfusion reserve; MPRI—myocardial perfusion reserve index; PET—positron emission tomography.

## Data Availability

Not applicable.
